# Patterns of Oral Microbiota Diversity in Adults and Children: A Crowdsourced Population Study

**DOI:** 10.1038/s41598-020-59016-0

**Published:** 2020-02-07

**Authors:** Zachary M. Burcham, Nicole L. Garneau, Sarah S. Comstock, Robin M. Tucker, Rob Knight, Jessica L. Metcalf, Genetics of Taste Lab Citizen Scientists, Anjelica Miranda, Brian Reinhart, Dani Meyers, Diane Woltkamp, Emma Boxer, Joyce Hutchens, Kelly Kim, Mike Archer, Mike McAteer, Phil Huss, Ravin Defonseka, Sean Stahle, Sunanda Babu, Tiffany Nuessle, Valerie Schowinsky, Wendy Covert, Weston Truman, Willy Reusser

**Affiliations:** 1 Department of Animal Sciences, Colorado State University, Fort Collins, CO 80525 USA; 2 Genetics of Taste Lab, Denver Museum of Nature & Science, Denver, CO 80205 USA; 3 Department of Food Science and Human Nutrition, Michigan State University, East Lansing, MI 48824 USA; 4 Department of Pediatrics, University of California San Diego, La Jolla, CA 92093 USA; 5 Center for Microbiome Innovation, University of California San Diego, La Jolla, CA 92093 USA; 6 Department of Computer Science and Engineering, University of California San Diego, La Jolla, CA 92093 USA; 7 Department of Bioengineering, University of California San Diego, La Jolla, CA 92093 USA

**Keywords:** Data processing, Metagenomics, Microbiome

## Abstract

Oral microbiome dysbiosis has been associated with various local and systemic human diseases such as dental caries, periodontal disease, obesity, and cardiovascular disease. Bacterial composition may be affected by age, oral health, diet, and geography, although information about the natural variation found in the general public is still lacking. In this study, citizen-scientists used a crowdsourcing model to obtain oral bacterial composition data from guests at the Denver Museum of Nature & Science to determine if previously suspected oral microbiome associations with an individual’s demographics, lifestyle, and/or genetics are robust and generalizable enough to be detected within a general population. Consistent with past research, we found bacterial composition to be more diverse in youth microbiomes when compared to adults. Adult oral microbiomes were predominantly impacted by oral health habits, while youth microbiomes were impacted by biological sex and weight status. The oral pathogen *Treponema* was detected more commonly in adults without recent dentist visits and in obese youth. Additionally, oral microbiomes from participants of the same family were more similar to each other than to oral microbiomes from non-related individuals. These results suggest that previously reported oral microbiome associations are observable in a human population containing the natural variation commonly found in the general public. Furthermore, these results support the use of crowdsourced data as a valid methodology to obtain community-based microbiome data.

## Introduction

Oral microbiome dysbiosis has been associated with various local and systemic human diseases including dental caries, periodontal disease, obesity, and cardiovascular disease^1–5^. Proper oral health care habits can help reduce abundance of taxa associated with pathogenic states. For example, flossing has been associated with decreased concentrations of the dental pathogen *Streptococcus mutans*^6^, and brushing of the teeth and tongue significantly decreases microbes associated with dental diseases^7,8^. It is estimated that over 600 bacteria species are commonly associated with the oral microbiome, with a subset of these proposed to be part of a consortium called the “core oral microbiome”^9–11^. Genera often considered associated with the core microbiome include *Streptococcus*, *Veillonella*, *Neisseria*, and *Actinomyces*, which are shared by most healthy individuals^12,13^. Maintaining the balance of core healthy bacteria in the oral microbiota plays a critical role not only in oral health but in overall health.

Oral microbiome composition is suspected to be affected by additional variables including host genetics^14,15^, geography^4,16^, diet^17,18^, age^19–21^, and cohabitation^22–24^. For example, comparative studies between European, African, Asian, and American populations discovered microbial variation between populations, and other studies describe ethnicity-specific clustering within the United States^15,25,26^. The effect of diet on oral microbiome composition was assessed through the discovery of bacterial shifts that occurred as human societies transitioned from a hunter-gatherer diet to more carbohydrate-rich diet associated with farming^17^. Currently, diets associated with Western industrialized societies have been shown to lead to poor oral health conditions, with bacterial relative abundances being affected by high sugar content; however, the full effects of the modern high carbohydrate and sucrose diet are still being elucidated^27,28^.

As individuals age, their oral microbiomes change and periodontal pathogens increase in abundance, leading to higher oral disease susceptibility^19–21,29^. Oral microbiomes of children are likely influenced by the increasing independence in oral health habits, transition to permanent dentition, and progression to an adult-like diet^18,20,29^. Interestingly though, children’s’ microbiomes continue to be similar to their parents even with independence^18,22^. Furthermore, the human tongue, skin, and stool human microbiomes have been shown to be more similar between cohabitating family participants than between individuals belonging to different families, suggesting a high level of microbial sharing between cohabiting individuals^14,22–24^. Shared environments have been shown to impact small-scale taxonomic differences in the human salivary microbiomes more than host genetics do^14,24^. These effects are likely established during upbringing and can persist for years in individuals who have moved out of the parental household; these findings support the hypothesis of a global core oral microbiome in which rare taxa are primarily affected by shared environments^24^.

To date, a majority of oral microbiome studies have been performed using controlled cohorts. Therefore, data resembling the natural variation found in a general population is still lacking^4,30–32^. Recently, the large-scale American Gut Project demonstrated the power of crowdsourced microbiome data for uncovering novel population-level microbiome trends^33^. We used a crowdsourced, cross-sectional convenience oral microbiome sampling of guests visiting the Denver Museum of Nature & Science in Colorado, USA to gain diverse, community-based data across age ranges, health statuses, and families for people living in an urban center of a Western industrialized country. Methods for the generation of crowdsourced data and utilization of citizen science at the Denver Museum of Nature & Science is outlined in Nuessle *et al*. (2020)^34^. Using this citizen science model, trained volunteer citizen scientists facilitated the collection from guests of buccal swabs for microbiome characterization. Additionally, guests provided information about oral health practices and status, family relationships, demographics, and beverage intake (both sugar-sweetened and unsweetened) to gain further understanding of the factors affecting the human oral microbiome. Height, weight, body mass index (BMI), and percent body fat were assessed. Additionally, participants were asked to taste a series of 5 sucrose solutions ranging from 0–13.7% w/v, as described in Garneau *et al*. (2018)^35^. Liking responses to these solutions were used to identify sweet-liking phenotypes. Sweet-liking phenotypes have been correlated with dietary intake of sugars and sugar-sweetened beverages, so associations between sweet-liking phenotype and oral microbiome composition were explored^35–37^. Employing a crowdsourcing data collection model provided a unique opportunity to test previously published and potentially novel characteristics that affect or are affected by oral microbiome composition within a general population^38,39^.

## Results

### Youth and adults differ in metadata proportions

A description of the cohort and sampling protocols can be found in the Methods. Chi-squared tests to compare metadata proportions between youth and adults determined multiple metadata factors to be significantly different (Table 1). Statistical tests were conducted using SAS 9.4 (Cary, NC). In brief, oral health metadata proportions that differed between age groups included time since last cavity, periodontal disease, time since last dentist visit, and brushing teeth daily. For instance, ~85% of adults have had a cavity compared to ~58% of youth, and ~9% of adults had periodontal disease compared to 0% of youth. Youth had also last visited the dentist more recently than adults. Adults and youth flossed with similar frequency, but adults brushed their teeth more often. Other demographic and anthropometric distributions that differed between youth and adults were age distribution, sex, BMI, percent body fat, and weight status. Adults had higher proportions of obesity, body fat, female participants, and greater age variance. Sweet liking phenotype categories and distribution also differed. Among adults, “likers”, “dislikers”, and “neutrals” were identified. Among youth, only “likers” and “dislikers” were identified. The youth population included more “likers” than the adult population.

**Table 1:** Metadata Proportion Comparisons Between Youth and Adults.

Metadata Category	Age Group		*p*-value
	Adult (n = 172)	Youth (n = 179)	
**Age, years (mean, median, range)**	(34.15, 33, 20–75)	(10.12, 10, 8–16)	<0.0001
**Male**	49 (28.49%)	71 (39.66%)	0.0301
**White**	146 (84.88%)	157 (87.71%)	0.4411
**BMI (mean, median, range)**	(27.15, 24.2, 17.2–51)	(17.65, 16.8, 13.5–30.7)	<0.0001
**% Body Fat (mean, median, range)**	(30.14, 29.5, 7.5–53.3)	(17.011, 15.5, 1.3–43.1)	<0.0001
**Last Cavity**			<0.0001
Never	26 (15.12%)	75 (41.9%)	
<3 mos ago	15 (8.72%)	9 (5.03%)	
3–6 mos ago	10 (5.81%)	13 (7.26%)	
6–12 mos ago	6 (3.49%)	17 (9.5%)	
1–2 yrs ago	28 (16.28%)	36 (20.11%)	
2 + yrs ago	87 (50.58%)	29 (16.2%)	
**Periodontal Disease**			0.0001
Yes	10 (5.81%)	0(0%)	
No	130 (75.58%)	162 (90.5%)	
Unsure	32 (18.6%)	17 (9.5%)	
**Last Dentist Visit**			<0.0001
<3 mos ago	52 (30.23%)	72 (40.22%)	
4–6 mos ago	42 (24.42%)	72 (40.22%)	
6–12 mos ago	29 (16.86%)	21 (11.73%)	
1 + yrs ago	49 (28.49%)	14 (7.82%)	
**Brush Teeth Daily**			0.0337
0–1×	40 (23.26%)	64 (35.75%)	
2×	117 (68.02%)	104 (58.10%)	
3x or more	15 (8.72%)	11 (6.15%)	
**Floss Teeth Daily**			0.3190
Never	80 (46.51%)	86 (49.04%)	
1×	79 (45.93%)	72 (40.22%)	
2x or more	13 (7.56%)	21 (11.73%)	
**Weight Status**			<0.0001
Normal	100 (58.14%)	144 (80.45%)	
Overweight	12 (6.98%)	21 (11.73%)	
Obese	60 (34.88%)	14 (7.82%)	
**Time Since Last Eating**			0.6584
<20 min ago	16 (9.30%)	23 (12.85%)	
20 min – 1 hr ago	36 (20.93%)	38 (21.23%)	
1–2 hr ago	56 (32.56%)	60 (33.52%)	
2 + hr ago	64 (37.21%)	58 (32.4%)	
**Abx In Last 6 Months**			0.8532
Yes	7 (4.07%)	8 (4.47%)	
No	165 (95.93%)	171 (95.53%)	
**Sweet Liker Status**			<0.0001
NA	0 (0%)	2 (1.12%)	
Likers	57 (33.14%)	134 (74.86%)	
Neutral	96 (55.81%)	NA	
Dislikers	19 (11.05%)	43 (24.02%)	

Chi-squared tests were used for metadata comparisons with significance determined by *p* < 0.05.

### Youth oral microbiomes are more diverse than adults

**Figure 1:**
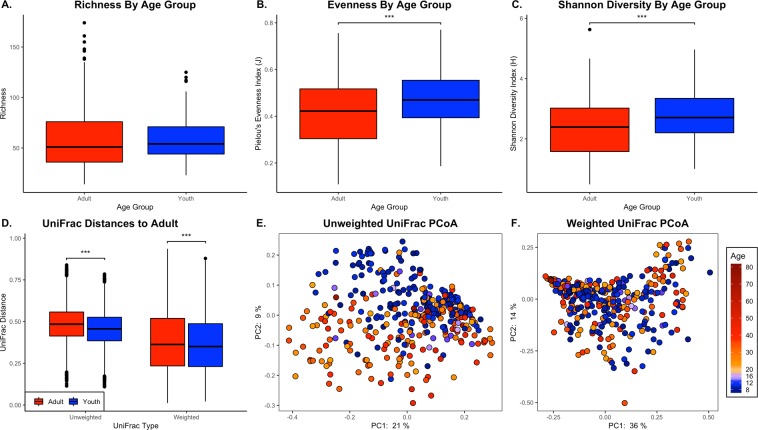
Adult and Youth Diversity Comparisons. Diversity comparisons between age groups using Kruskal-Wallis tests on (**A)**. Richness, (**B)**. Evenness, and (**C)**. Shannon’s index. PERMANOVA tests were used to compare (**D)**. UniFrac distances, which were visualized as (**E)**. unweighted and (**F)**. weighted UniFrac PCoA plots. UniFrac distance comparisons were as adults to adults and adults to youth. Significance determined by *q* < 0.05 (*), ≤0.01 (**), ≤0.001 (***).

When comparing the youth and adult oral microbiomes, median richness was not statistically significantly different, but trended higher in youth samples (Fig. 1A). Youth samples had higher median evenness (H = 14.5, *q* = 1.0 × 10^–4^) and Shannon diversity (H = 12, *q* = 6.0 × 10^–4^) (Fig. 1B,C). Weighted and unweighted UniFrac distances differed between adults and youth (Unweighted: N = 351, pseudo-F = 12.5, *q* = 1.0 × 10^–3^; Weighted: N = 351, pseudo-F = 7.3, *q* = 1.0 × 10^–3^) (Fig. 1D). Youth diversity metrics had less intra-group variation as compared to the adults due to the adult samples having higher variability in Shannon, evenness, richness, and UniFrac metrics. Visualizing the UniFrac distances in a Principal Coordinate Analysis (PCoA) plot shows greater separation by age group when using unweighted metrics versus weighted metrics (Fig. 1E,F).

**Figure 2:**
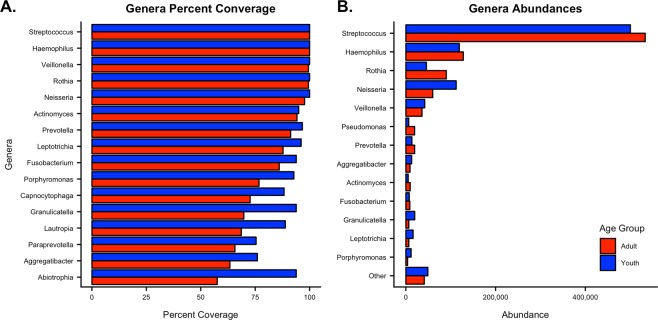
Adult and Youth Genera Compositions. (**A)** Genera percent coverage and (**B)** abundances are shown in descending order by adult samples. Genera percent coverage is represented as the percent of samples in which a genus was detected and was only calculated if a genus was present in at least 75% of samples in either age group. Genera abundances were calculated for each age group based on read counts.

Top genera coverages and abundances show adult and youth oral microbiomes are primarily dominated by few taxa (Fig. 2). Youth have more high-coverage (≥75%) genera than adults (Fig. 2A). *Streptococcus* and *Haemophilus* were detected in 100% of adults with *Veillonella*, *Rothia*, and *Neisseria* detected 99.4%, 99.4%, and 97.7% of the time, respectively. All five genera were found in 100% of youth samples. When examining the genera abundances between the age groups, *Streptococcus* dominates the microbial landscape with 54% and 44% of the total abundances in adults and youth, respectively (Fig. 2B). Including the top coverage genera, the oral microbiome is dominated by *Streptococcus*, *Haemophilus*, *Rothia*, *Neisseria*, and *Veillonella*, which make up 85.4% of all adult genera and 71.7% of youth.

Analysis of composition of microbiomes (ANCOM) determined 12 genera to be differentially abundant between the age groups (Table 2). The three most significant differentially abundant genera were *Abiotrophia* (W = 133, F = 107.3), *Granulicatella* (W = 133, F = 67.3), and *Treponema* (W = 131, F = 52.0). *Abiotrophia* had the largest effect size difference and was found more often in youth (median = 48.0, max = 1217.0). *Treponema* was the genus with the largest effect size difference that was found more often in adults (median = 1.0, max = 2116.0).

**Table 2:** Differentially Abundant Genera between Adults and Youth.

Genera	Median Percentile Abundance		Max Percentile Abundance		W	F
	Adult	Youth	Adult	Youth		
*Abiotrophia*	5.5	48.0	478.0	1217.0	133	107.3
*Granulicatella*	16.0	152.0	1345.0	1863.0	133	67.3
*Treponema*	1.0	1.0	2116.0	78.0	131	52.0
*Porphyromonas*	14.0	54.0	890.0	1712.0	125	42.8
*Capnocytophaga*	9.5	27.0	402.0	1866.0	122	39.7
*Lautropia*	8.0	19.0	1423.0	866.0	122	35.4
*Neisseria*	235.5	546.0	14752.0	9859.0	121	26.5
*Leptotrichia*	25.0	54.0	899.0	2793.0	116	22.0
*Atopobium*	1.0	1.0	82.0	34.0	113	41.8
*Megasphaera*	1.0	1.0	289.0	27.0	110	41.6
*Cardiobacterium*	1.0	1.0	40.0	426.0	106	15.7
*Actinobacillus*	1.0	11.0	2365.0	4869.0	104	14.1

ANCOM determined significant differential genera abundances with their 50th percentile abundance (median), highest sequence count found in a sample (max), W-statistic and F-score. W-statistics represent the strength of the ANCOM test, and F-scores represent the effect size difference of that genera between groups.

### Adult oral microbiomes are affected by oral health habits

**Figure 3:**
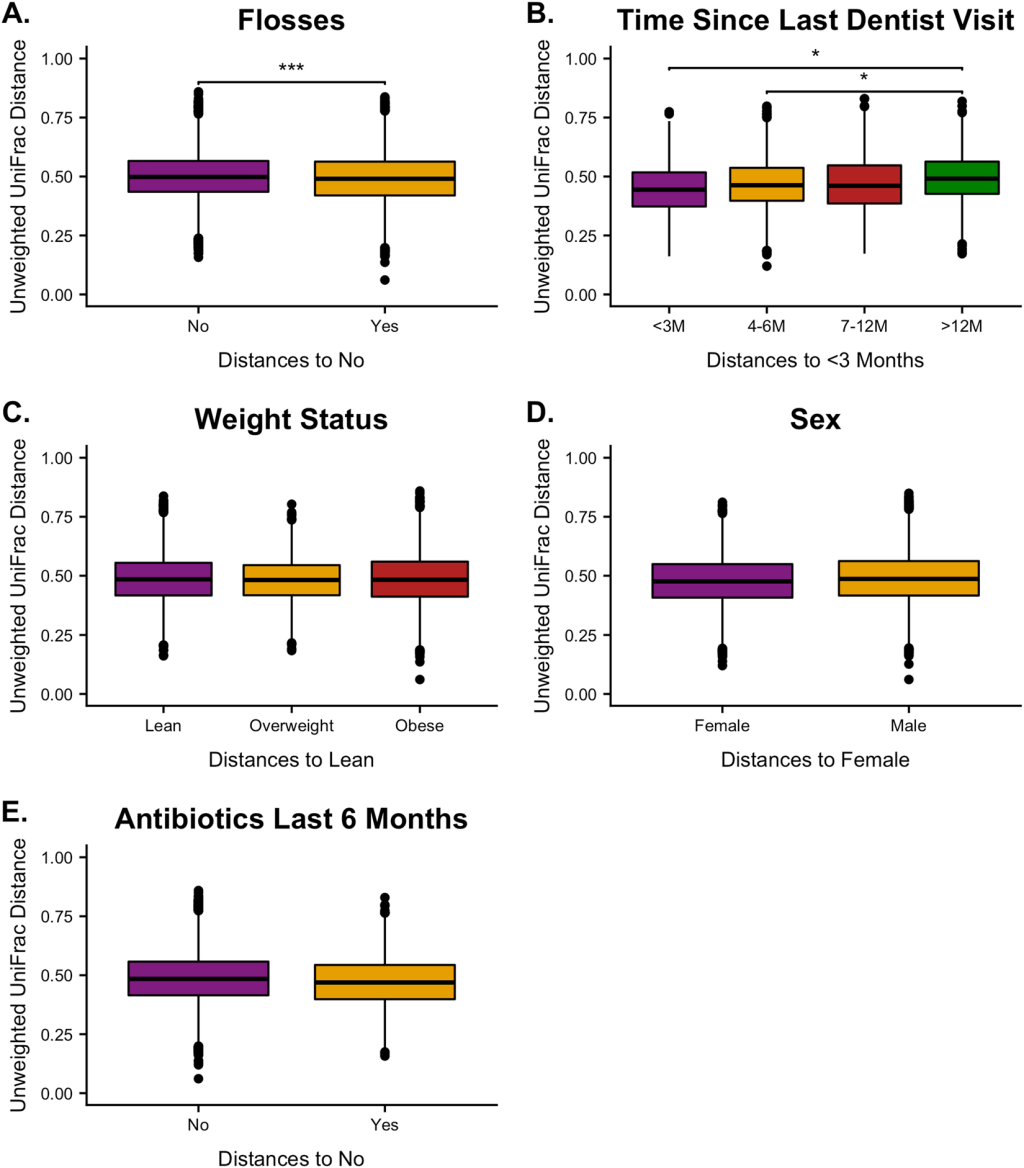
Beta Diversity Comparisons in Adults. Unweighted UniFrac adult distance comparisons on (**A**). flosses, (**B)**. time since last dentist visit, (**C)**. Weight status, (**D)**. Sex, and (**E)**. Antibiotic use last 6 months using PERMANOVA. Significance determined by *q* < 0.05 (*), ≤0.01 (**), ≤0.001 (***).

Metadata factors with previously reported or suggested associations to the oral microbiome were compared within adult samples using unweighted UniFrac distances (Fig. 3). For example, flossing significantly altered beta diversity (Fig. 3A). To determine if flossing frequency affected the oral microbiome, unweighted UniFrac distances were compared by the number of times an individual flossed each day. Only non-flossers and individuals flossing once per day were considered significantly different (N = 159, pseudo-F = 3.5, *q = *0.03) although twice daily flossers were just beyond the significance threshold (N = 92, pseudo-F = 2.3, *q = *0.06). We believe this may be due to the smaller number of individuals flossing multiple times a day which created a sample size too small to accurately observe a trend. An expanded study will be needed to draw conclusions on the impact of daily flossing frequency. For this reason, we focused on just measuring if an individual was a flosser or non-flosser. Compared to non-flossers, individuals who reported flossing regularly had a lower median UniFrac distance than non-flossers (N = 172, pseudo-F = 4.2, *q = *1.0 × 10^–3^). ANCOM did not determine any genera to have significant differential abundance between flossing groups. Adult beta diversities also differed based on time since the individual’s last dentist visit (Fig. 3B). Unweighted UniFrac distances increased in a stepwise pattern as the time since previous dentist visit increased when compared to the less than 3 months group. Pairwise comparisons showed adults who had been to the dentist within 3 months of being sampled compared to the adults who had not been in over 12 months had the greatest differential (N = 101, pseudo-F = 2.8, *q = *0.04), followed by the 4-6-month group compared to the over 12-month group (N = 91, pseudo-F = 2.59, *q = *0.04). ANCOM determined *Treponema* to be differentially abundant across timepoints and detected more in the over 12 months group (W = 106, F = 8.8). Adult oral microbiomes did not differ when comparing weight status, sex, or antibiotic usage over the last 6 months (Fig. 3C–E).

### Youth oral microbiomes are affected by sex and weight Status

**Figure 4:**
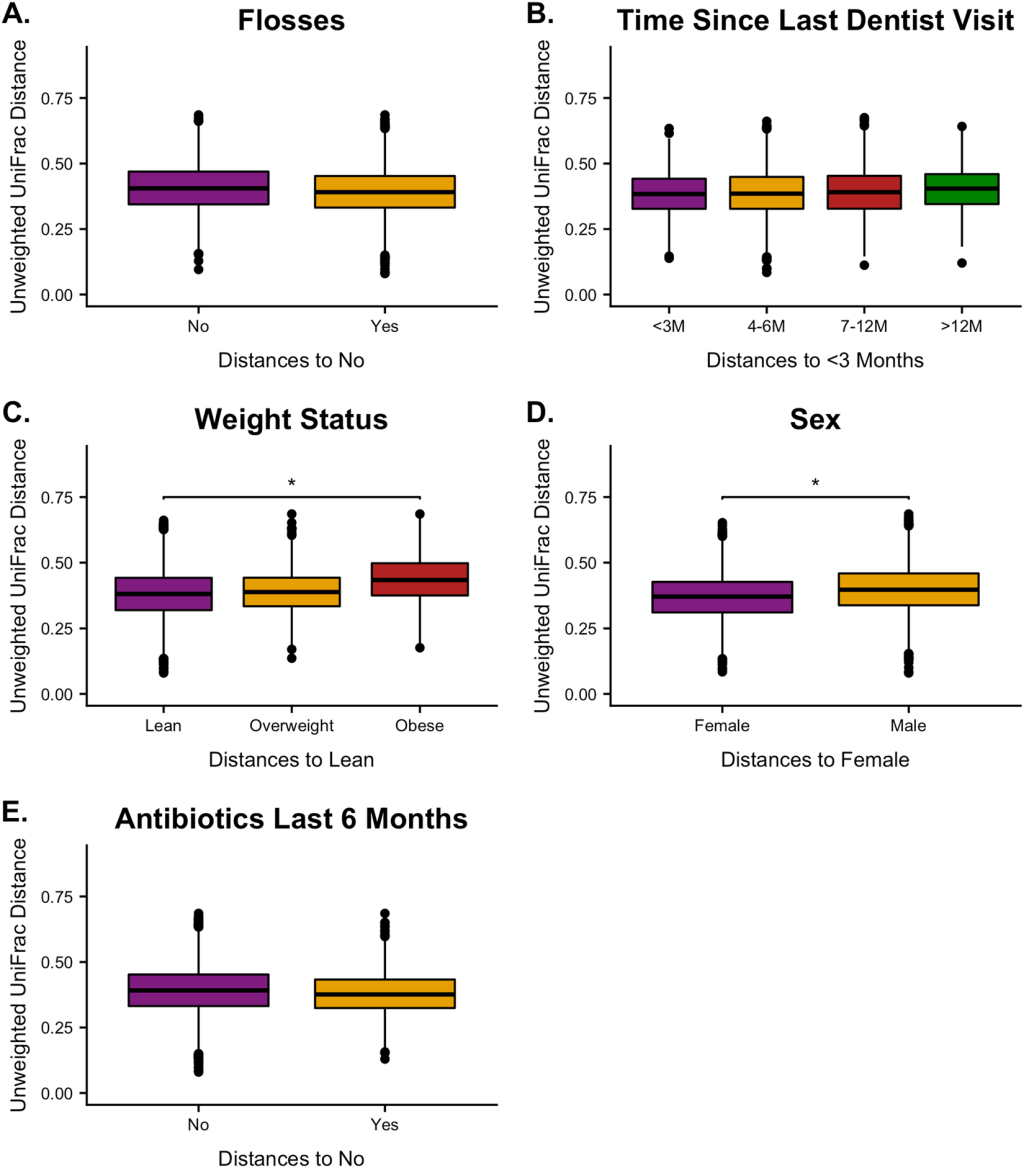
Beta Diversity Comparisons in Youth. Unweighted UniFrac youth distance comparisons on (**A)**. flosses, (**B)**. Time since last dentist visit, (**C)**. Weight status, (**D)**. Sex, and (**E)**. antibiotic use last 6 months using PERMANOVA. Significance determined by *q* < 0.05 (*), ≤0.01 (**), ≤0.001 (***).

The same metadata factors analyzed in adults were compared within youth samples using unweighted UniFrac distances (Fig. 4). Youth distances showed similar patterns to adults in flossing frequency and time since last dentist visit, but were not significant (Fig. 4A,B). Distances increased as youth weight statuses increased from lean to overweight and obese when compared to the lean group, but the overall model was not significant (Fig. 4C). However, pairwise comparisons showed a significant difference between youth who were considered lean compared to those considered obese (N = 158, pseudo-F = 2.5, *q = *0.04). ANCOM determined *Treponema*, the genera associated with prolonged time between dentist visits in the adult group, to be differentially abundant between weight statuses with increased detections in obese youth (W = 55, F = 10.2). Since weight status was associated with changes in oral microbiome, BMI was additionally tested using the weighted and unweighted UniFrac distances in a Spearman correlation Mantel test. BMI was found to have a positive correlation with both unweighted and weighted UniFrac measurements (Unweighted: N = 179, *r*_*s*_ = 0.10, *p* = 3.0 × 10^–3^; Weighted: N = 179, *r*_*s*_ = 0.07, *p* = 0.03). Youth males had a higher median distance than females when compared to females (N = 179, pseudo-F = 2, *q = *0.03), though no specific genera were determined to be differentially abundant via an ANCOM analysis (Fig. 4D). Youth oral microbiomes did not differ when comparing antibiotic usage over the last 6 months (Fig. 3E).

### Participants from the same family have more similar oral microbiomes

**Figure 5:**
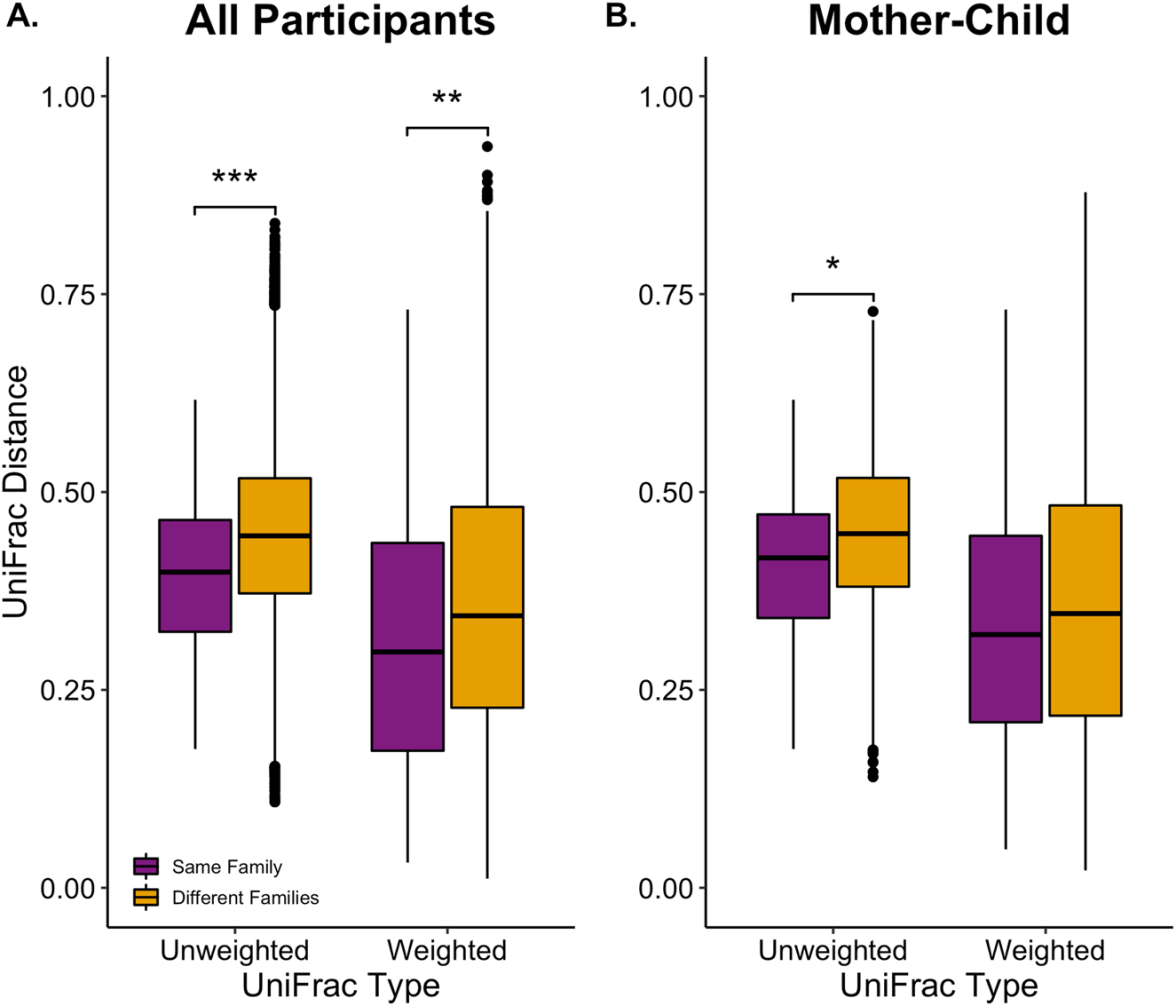
Familial Beta Diversity Comparisons. Wilcoxon rank-sum test comparing unweighted and weighted UniFrac distances of (**A)**. All participants within the same family against all participants of different families and (**B)**. Same family mother-child distances against different family mother-child distances. Significance determined by *q* < 0.05 (*), ≤0.01 (**), ≤0.001 (***).

Overall, participants from the same family had more similar oral microbiomes than individuals belonging to different families (Fig. 5). There were 163 within family and 61,262 between family distance comparisons. When comparing all within family to all between family distances, both unweighted and weighted UniFrac distances differed (Unweighted: W = 3.6 × 10^6^, *q = *6.6 × 10^–9^; Weighted: W = 4.0 × 10^6^, *q = *1.1 × 10^–3^) (Fig. 5A). Twin siblings were found to be no different than other within-family sibling types. Since mother-child relationships have been shown to affect microbiomes, within family mother-child distances were compared to between family mother-child distances, and only unweighted UniFrac distances were significantly different (Fig. 5B). There were 74 within family mother-child and 3,571 between family mother-child distance comparisons.

### No oral microbiome differences in adults or youth based on sweet-liking phenotype or beverage consumption

**Figure 6:**
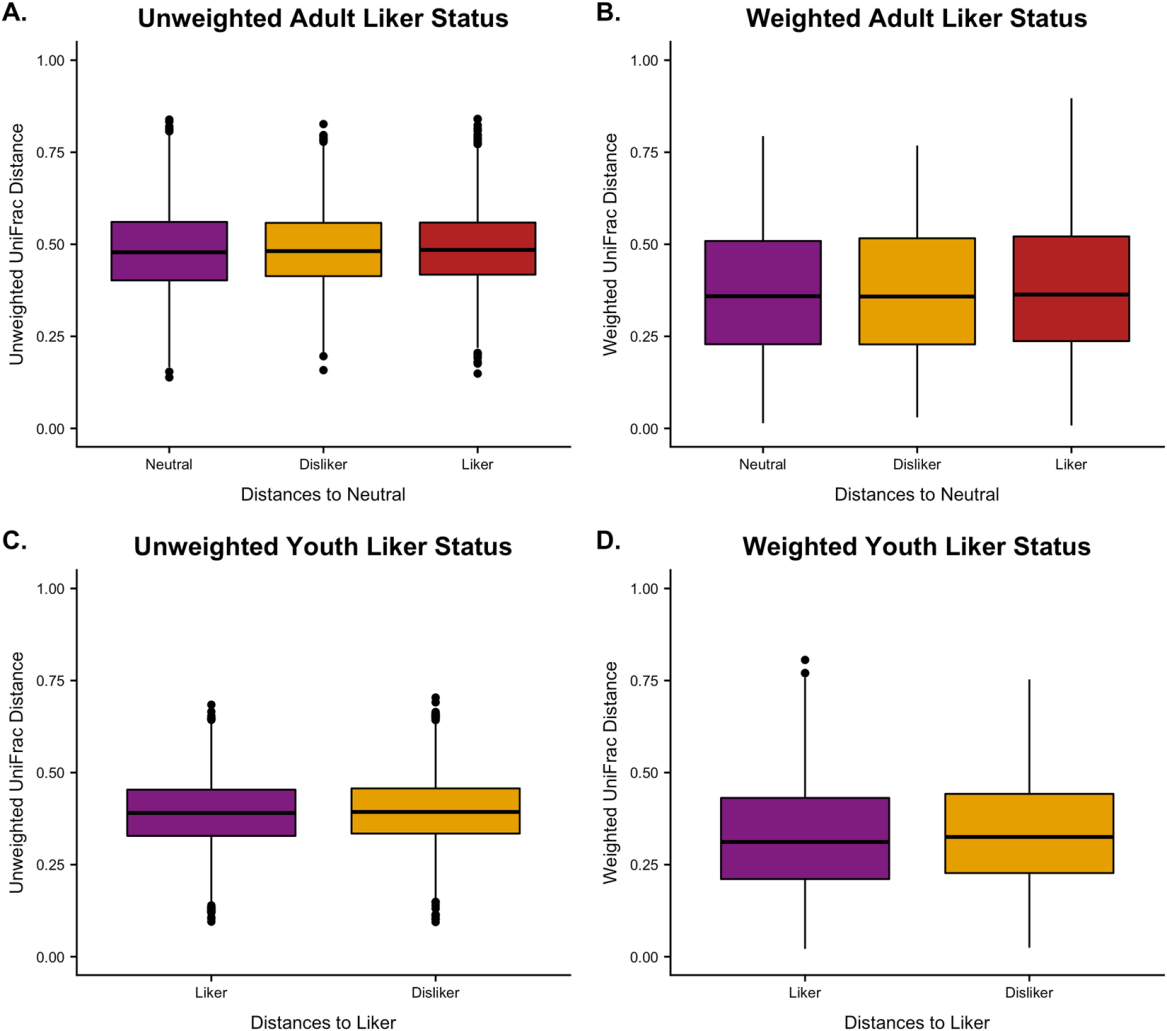
Beta Diversity Comparison on Sweet-Liking Phenotype. UniFrac distance comparisons on (**A)**. adult unweighted distances, (**B)**. Adult weighted distances, (**C)**. Youth unweighted distances, and (**D)**. Youth weighted distances against sweet-liking phenotype using PERMANOVA. Significance determined by *q* < 0.05 (*), ≤0.01 (**), ≤0.001 (***).

Previously, hierarchical cluster analysis identified several distinct sweet-liking phenotypes in both children and adults^35^, and because taste preferences change as we age, adults and youth were analyzed separately^35^. In this study, no associations with the oral microbiomes of adults or youth and sweet-liking status were detected (Fig. 6). Additionally, adult oral microbiome associations with sugar and alcohol beverage energy (kcal) intake were not detected. Beverage consumption data was not collected for youth.

## Discussion

The goal of this study was to characterize the oral microbiome of a general population in the United States using a citizen-scientist model. We used crowdsourced bacterial community data to characterize relationships between microbial composition and individual demographics, oral health attributes, and sweet-liking phenotype. Our data suggests adults and youth share central core oral microbiome genera, but vary in rare taxa abundance and coverage (Fig. 1), where rare taxa were considered genera that constitute less than 3% of the community composition. Youth had higher median alpha diversity than adults, although adults had a higher diversity distance range. Youth also had a lower median UniFrac distance when compared to adults than when adults were compared to each other. This is likely due to youth microbiomes being more similar to each other compared to adults which had higher intra-group (person-to-person) variation and higher dissimilarities (i.e. more personalized microbiomes). Additionally, youth microbiomes contained more genera detected in over 75% of the population (16 in youth compared to 10 in adults) (Fig. 2A) and higher abundances of rare taxa as represented by higher count taxa constituting less than 3% of the community composition such as *Granulicatella*, *Leptotrichia*, and *Porphyromonas* (Fig. 2B). These data indicate that youth oral microbiomes are more diverse than adults as a population, but adult communities have more intra-group variation. These results are largely in agreement with a study by Ling *et al*. (2013) which compared the salivary microbiomes of healthy Chinese adults and children^40^.

Although some genera associated with the core oral microbiome, such as *Prevotella*, varied in abundance across our two populations, they still maintained high coverage (≥75%) (Fig. 2). The presence of these genera in nearly all samples across two distinct populations strengthens the hypothesis of a global core oral microbiome, at least within urban United States, and suggests environmental and health factors may contribute to genera abundances but may not necessarily eliminate core genera from being present at low levels. Currently, it is difficult to assess which specific variables are causing the differences between populations, but previous research suggests culture and sampling locations are likely contributors^4,16,25^. Twelve genera were differentially abundant between age groups, and are all known inhabitants of the oral cavity (Table 2). Many of them are associated with normal, healthy oral microbes, but some are associated with oral and/or systemic diseases when in dysbiosis^4,5,12^. Increased abundances of *Abiotrophia* and *Granulicatella*, which were found more often in youth communities, have been associated with dental caries, obesity, and endocarditis^40–44^. *Treponema* was more often found in adult communities; a genus commonly known for its role in the “red complex” associated with severe forms of periodontal disease and dental caries^45^. Additionally, *Treponema* abundance increases have been associated with increasing age^29^. This may be indicative of differences between the effects of adult and youth habits, diet, and healthcare quality and frequency. Comparing the metadata proportions between age groups further strengthens this idea as adults had statistically higher proportions of oral health issues, less frequent dentist visits, and increased prevalence of obesity (Table 1).

When separating the two age groups and analyzing them individually, we found that each group’s microbiome variation was driven by different factors. The adult oral microbiomes were affected by flossing and time since last dentist appointment (Fig. 3). Previous work comparing twin flossers vs. non-flossers observed periodontal pathogens were overabundant in non-flossers while healthy microbiota were overabundant in flossers over a two-week study^7^. In the adults of our study, flossers have reduced beta diversity variation potentially caused by the disruption of small niches and physical removal of rare taxa during the flossing process leaving higher relative abundances of core oral microbiome genera behind or by invoking inflammation and activation of the immune system (Fig. 3A). The positive correlation in adults between UniFrac distances and time since last dentist visit suggests dentist visits reduce natural variation and diversity in the oral microbiota, but this variation is gradually reintroduced (Fig. 3B). The oral pathogen *Treponema* was associated more often with individuals who had not been to the dentist in over 12 months. It is likely, as with flossing, dental cleanings physically remove rare taxa, invoke inflammation, and activate the immune system leading to reduced oral microbiota diversity while helping control pathogen overabundance. Previously, species richness of biofilm redevelopment after dental cleaning has been shown to be increased in periodontitis-affected individuals^46^. A future in-depth study analyzing oral microbiomes before and after dentist visits with long term longitudinal sampling would be beneficial to determine overall variation and the specific genera that are lost during dental cleanings and if variation reintroduction is caused by the remaining traces of previously present or newly introduced genera.

Youth oral microbiomes were less affected by oral health care habits than adult oral microbiomes (Fig. 4). Youth reported flossing habits similar to adults, but unexpectedly did not show the same microbiome changes (Fig. 4A). This raises the question of whether youth oral microbiota are more resilient to diversity loss due to their increased relative abundances of rarer taxa and higher taxa evenness. Flossing has been determined to affect the oral microbiomes of teenagers, but the effects of flossing on younger children is still unknown^7^. In this study, ~87% of the youth were 8–9 years of age which suggests flossing may alter diversity differently in prepubescent children. It is also possible youth are more likely to misrepresent how often they floss and may even perform flossing inadequately as compared to teen or adult flossers. Therefore, it is difficult to assess whether the reduced effect of flossing with prepubescent children is correlated to microbial ecology or self-reported data. Youth oral microbiome diversity changed similarly to adults with time since last dentist visit but was not significant (Fig. 4B). This may be due to the fact that more youth had recently been to the dentist, while more adults had not been to see a dentist recently. For example, ~80% of youth had been to the dentist within 6 months and only ~8% had not been to the dentist in over 12 months compared to adults which only ~54% had been to the dentist within 6 months and ~29% had not been to the dentist in over 12 months (Table 1). This made it difficult to appropriately assess the impact of the time since last dentist in youth oral microbiomes.

Gut and oral microbiota dysbiosis has been detected in obese adolescents and children^3,44,47^. Recently, young children with a high risk of childhood obesity have been associated with the loss of oral microbiota diversity over early growth with microbiota resembling obese adults by age two^31^. Our study only included youth at least 8 years of age, predominantly children 8–9 years old. Our data show oral microbial communities to be distinct between lean and obese youth with no specific genera being differentially abundant (Fig. 4C), and we observed obese youth to have increased phylogenetic diversity, as opposed to decreasing overall diversity as previously reported^31^. We also found BMI metrics to have a positive correlation with both unweighted and weighted UniFrac distances. Craig *et al*. (2018) describe an immediate loss of oral alpha diversity in young children over the first 10 months of growth, but diversity begins to increase again by 25 months as oral microbiota associated with obesity are more prevalent^31^. This increase in alpha diversity may continue into prepubescent childhood as we see increased diversity in our data, but further longitudinal studies between 2 and 8 years of age are needed. Interestingly, *Treponema* was determined to be found more often in obese youth, which is the same genera associated with poor oral health found in the adult group. In adults, obesity and poor oral health has been linked with obese individuals having higher prevalence of periodontitis^48,49^. These data suggest that this link may be observable in youth as well.

Additionally, youth male and female oral microbiomes were determined to be different, but no specific genera were determined to be the cause (Fig. 4D). It is unclear why youth male and female microbiomes differ while adult male and female did not. Possible causes include less attentive hygiene habits, and that standards of cleanliness are generally higher for young females than males^50^. One limitation to this study is that we cannot consider the impact of puberty or hormones. However, the majority of youth participants were likely prepubescent, and we found no significant differences in the oral microbiomes of adult males and females supporting a limited effect of hormones on the oral microbiome.

Cohabitation and familial relationships have been shown to increase oral microbiome similarity more than host genetics, in particular among rare taxa^22,24^. Our data suggest this impact on diversity is still observable within a general population (Fig. 5). Participants of the same family have a greater similarity to each other over participants of different families (Fig. 5A). This was also found, albeit to a lesser extent, in mother-child pairs (Fig. 5B). Unsurprisingly, unweighted UniFrac distances were more sensitive to inter-family comparisons than weighted UniFrac distances potentially due to rare taxa variation across families that are not part of core oral microbiome. Same family distances also consistently contained less variation in similarity, suggesting intra-family relationships and cohabitation leads to the sharing of both core and rare taxa^24^. Weighted differences were still detected between all participants of the same families to different families even though most individuals shared a core set of microbes, which suggests core microbiota differ in abundance across families while still maintaining high coverage within the population.

Sweet-liking phenotypes differed between children and adults. Sweet-liking phenotypes identified for children were “likers” and “dislikers”, while adult phenotypes also included an additional phenotype of “neutral”^35,37^. These sweet-liking phenotypes are not associated with sex, age, or BMI, but adult sweet “likers” did consume more sweet beverages than “neutral likers”^35^. The concept of the oral microbiota contributing to food choice and taste perception is currently being investigated since similar associations have been found with the gut microbiota^51^. One recent study examined relationships between taste perception, food intake, and oral microbiota composition in adults^52^. The authors reported that bacteria classes, such as Prevotella and Clostridia, were associated with the taste perception of vegetable-rich and protein-rich diets. Additionally, decreased Clostridiales Family XIII relative abundance was the only taxa associated with sweet taste thresholds in adults. Taste sensitivity, as measured by thresholds, are not frequently associated with hedonic evaluations or dietary behaviors, so it is not altogether unexpected that this study did not identify microbiome composition associations with sweet-liking phenotypes or energy intake from beverages (Fig. 6)^53^. Further work is needed to explore what role the oral microbiome plays on taste sensitivity and perception, food selection, and dietary intake.

In this study, a model of crowdsourcing was used to test oral microbiome associations in a general population. We found that adult and youth oral microbiomes share core taxa, but are distinct from each other in taxa abundances and coverage. Factors affected each age group differently with adults largely impacted by oral health habits, and youth impacted by weight status. The oral pathogen genera *Treponema* was associated with both poor oral health habits in adults and obesity in youth. Additionally, we found oral microbiomes from participants of the same family were more similar to each other than to oral microbiomes from unrelated individuals. While a number of biological factors were compared, most had no statistical significance, and those that were statistically significant, such as weight status and flossing, appeared to differ minimally. We believe that the detection of these effects in a general population demonstrates that the selective pressures applied to the oral community from each factor are strong enough to overcome interpersonal variations and other background confounding variables, such as genetics, lifestyle, and living situation. Importantly, the small variations detected can be attributed to few select taxa, such as *Treponema*, which are considered pathogenic and associated with multiple health risks. This leads to seemingly specific oral microbiome differences potentially contributing to major health complications, such as periodontal disease and cardiovascular disease^1,2,4,5^.

Nonetheless, our study has certain limitations. Firstly, the reduced range of age in the youth population as compared to the adults may limit age comparison conclusions. Secondly, youth are in the transition period of temporal to permanent dentition which may increase inflammation, microbial niches, and create unequal tooth presence/absence across this group. Additionally, the participants sampled in this study were limited to a Western industrialized population living in an urban center, and thus conclusions may not be globally generalizable^15–17^. However, these results further suggest a connection of the oral microbiome to human health, particularly obesity. Lastly, due to the nature of this cross-sectional study, all potential exposures and confounding variables that can affect the microbiome could not be measured (e.g. individual diet, habitat, medications, etc.).

This study was aimed as both a scientific study and a visitor participation experience which engages the participants to “create content” and gather a better understanding of scientific methods^34,54,55^. These results are relevant to the scientific community because they demonstrate (similar to the American Gut Project) that important human microbiome trends can be recovered using a citizen science model, which can be a powerful and cost-effective means for collecting from a large population, and engages the public in science^33^. Crowdsourcing in the Genetics of Taste Lab at the Denver Museum of Nature & Science consisted of two forms of community contribution to the scientific process: (1) guests enhanced their science education experience by participating in the study as human research subjects, and (2) enabled volunteer citizen scientists to obtain training, collect data, and support in data analyses.

## Methods

### Participants and sample collection

Participants ages 8 and older were recruited from guests to the Denver Museum of Nature & Science, between November 2015 and August 2016 (N = 351). The Denver Museum of Nature & Science was chosen because they have a Genetics of Taste Lab on the premises and have an established record of successful crowdsourcing projects^35,38,56–58^. This community lab is uniquely situated to attract both a large population of human subjects and host a team of citizen scientists to research population-based questions about human genetics, taste, and health^38^. Participants provided informed assent or consent for participation in the Sweet Tasting Study in the Genetics of Taste Lab, and parental permission for children between the ages of 8–17. The only exclusion criterion was age – children under the age of 8 were not eligible. The study was approved by The Bowling Green State University Human Subjects Review Board (approval # 796133) and complied with the Declaration of Helsinki for Medical Research involving Human Subjects. Informed consent was obtained from all subjects, and if subjects were under the age of 18 years, their legal guardian provided informed consent and was required to be present.

The participant sample population consisted of a total of 366 individuals with 181 adults and 185 youth. Of this group, 15 individuals were excluded from the study due to poor sequencing depth. The final analysis was performed on 351 individuals (N = 351) with adults = 172 (n = 172), youth = 179 (n = 179). Height, weight, and percent body fat were measured using a freestanding stadiometer and bioelectrical impedance analyzer (Tanita TBF-215, Tanita, Tokyo, Japan). Body mass index (BMI) was calculated in both children and adults, with children’s BMI converted to z-scores to normalize across age, sex, and height^59^. Children were designated as lean, overweight, or obese based on the z-score classification of the World Health Organization^60^.Individuals younger than 20 years old were considered youth based on the Centers for Disease Control and Prevention recommended age separation for BMI percentile calculation (cdc.gov/healthyweight/assessing/bmi/index). Adults were, on average, 34.2 years old (min = 20, max = 75, median = 33) and youth were, on average, 10.1 years old (min = 8, max = 16, median = 10). Adult BMI calculations were performed using the formula: *weight(kg)/[height(m)]*^2^, while youth BMI z-scores were calculated using the Stanford Children’s Health calculator which accounts for the individual’s sex and age (stanfordchildrens.org/en/topic/default?id = childrens-bmi-calculator-41-ChildBMICalc). The entire list of sample metadata can be found as Supplementary Table S1.

In order to obtain a general oral microbiome sample, participants were each given an Epicentre Buccal Swab. Once shown how to open, use, and securely replace the swab back into the transport system, participants were instructed to scrub for 30 seconds all over the inside of their mouths, including teeth, tongue, cheeks, and gums. Swabs were stored at −20 °C until DNA extraction.

### Sweet-liking preference and energy consumption determination

Sweet-liking phenotype refers to individual differences in sweetness preference as concentration varies^37^. Sweet “likers” rate increasing concentrations of sweetness as increasingly liked, sweet “dislikers” rate increasing concentrations of sweetness as increasingly disliked, and “neutral” individuals provide consistent liking ratings regardless of sweetness concentration. For this study, classification of sweet liker status was previously described by Garneau *et al*. 2018. Briefly, sweet taste intensity and liking measures were conducted in a randomized, double-blind manner. Participants sampled 5 mL by the swishing and spitting of five concentrations of sucrose dissolved in deionized (DI) water (0.0% (blank), 2.4% (low), 4.3% (medium), 7.7% (high), 13.7% (highest) w/v). Sweet taste intensity and liking for each solution was performed using 100 mm visual analog scales (VAS) with the anchors: ‘extremely weak,’ and ‘extremely strong’ and ‘dislike extremely’ and ‘like extremely’, respectively. Participants worse nose clips were during taste testing and rinsed with bottled water between each sample. Hierarchical cluster analysis was used to identify three sweet liking patterns in adults and two patterns in children. In both adults and children, participants who reported increased liking as concentration increased were classified as “likers” and participants who reported decreased liking as concentration increased were classified as “dislikers”. In adults, a third group was classified as “neutrals” because liking scores remained relatively constant regardless of concentration. Previous work reported increased intake of total sugar^36^, refined sugar^36^, and sugar sweetened beverages^35,61^, with “likers” consuming more. Since these dietary differences could lead to differences in oral microbiota, relationships between microbial composition and sweet-liking phenotypes were explored.

Energy consumption from beverages was determined as previously described^35^. Briefly, adults completed the BEVQ-15, a validated beverage food frequency questionnaire^62^. The BEVQ-15 asks how much (ounces) and how often (times per day) various common beverages are consumed (e.g. water, coffee, soft drinks). Based on the amount and frequency of consumption, energy intake was determined using the BEVQ-15 protocol^63^.Total energy intake was calculated by adding the contribution of all beverages. Energy contributions from sweetened juice, sweetened tea, regular soft drinks, tea and coffee with cream and/or sugar, and energy drinks determined energy intake from sugar-sweetened beverages. Because some individuals take coffee with cream but not sugar, and due to the fact that energy drinks are available in both sugar-containing and sugar-free varieties, these categories were excluded from microbiome analyses.

### DNA Extraction and 16S rRNA gene amplicon sequencing

DNA was extracted from Epicentre Buccal Swabs and purified using the Maxwell 16 Buccal Swab LEV DNA Purification Kit (Promega AS1295) and the Promega Maxwell 16 (AS1150), set on the standard bacterial protocol extraction setting for the machine. Purified DNA samples were then loaded into the wells of 96 well plates at 30 µl and a minimum of 10 ng/µl per sample. We left four blank wells for sequencing controls and the plates were sealed and stored at −20 °C. Samples were shipped on dry ice to the Knight Lab at the University of California San Diego for amplification and sequenced using an Illumina MiSeq, targeting the V4 region of the 16S rRNA gene using modified 515F–806 R primers as recommended by the Earth Microbiome Project^64^. The V4 region was also chosen based on its nearly universal bacterial and archaeal annotation and availability for alignment in reference databases such as Greengenes^65^.

### Amplicon sequencing data processing

Sequencing data and sample metadata were uploaded to the QIITA open-source microbiome study management platform under study 11293^66^. QIITA serves as a multi-omics data repository capable of utilizing the QIIME2 processing pipeline^66,67^. Within QIITA, sequence reads were demultiplexed, trimmed in length to 150 bp, and deblur v.1.1.0 was used with default settings to quality filter and create amplicon sequence variants (ASVs) based on the Greengenes 13.8 reference phylogeny using SEPP fragment insertion^68–71^. Default settings include a mean per nucleotide error rate of 0.005, insertion/deletion (indel) probability of 0.01, and maximum number of indels at 3. The deblur pipeline performs *de novo* chimera filtering using UCHIME as implemented by VSEARCH and rapidly uses error profiles in a sensitive manner to obtain putatively true, high quality biological sequences^71^. Deblur ASV determination was used over traditional OTU-picking methods due to the loss of sequence variation that occurs when sequences are collapsed at a specific sequence identity percentage in attempts to obtain species-level clustering^69,71^. SEPP fragment insertion performs a phylogenetic placement technique explicitly designed for 16S rRNA data to obtain improved phylogeny trees^70^. The ASV feature table containing hits to the Greengenes 13.8 reference database, metadata, and reference-hit representative sequences were imported into the QIIME2 v.2019.1 microbiome bioinformatics platform^67^.

### Taxonomy assignment and diversity metrics

Taxonomy was assigned using a Greengenes 13.8 16S rRNA V4 region classifier at 99% sequence identity^68,72^. The Greengenes database was utilized because it is chimera-checked and tailored for 16S rRNA classification^68,72^. When performing 16S rRNA alignment, SILVA and Greengenes map comparable to NCBI^73^. Sequences filtered out of the feature table included those assigned to mitochondria and chloroplast along with features present less than 10 times to reduce noise. Samples belonging individuals unable to be classified to the adult or youth groups were excluded. Rarefaction was performed at a depth of 6,380 sequences to remove poorly sequenced samples, sequencing negative controls, and provide even sampling across the remaining samples for diversity metrics. In addition to the filtered table containing all remaining features, tables were generated collapsed at different taxonomic levels including: phylum, class, order, family, genus, and species.

The QIIME2 diversity plugin was used to compute the following alpha and beta diversity metrics: Shannon’s diversity index (H), Pielou’s evenness index (J), observed ASVs (richness), Faith’s phylogenetic diversity (phylogenetic richness), and weighted and unweighted UniFrac distances^67,74^. UniFrac distances were visualized as principal coordinate analysis (PCoA) ordination plots using EMPeror^75^. Diversity metrics were calculated for the population as a whole along with adult and youth samples grouped separately. This was performed to allow diversity metric comparisons between and within each age group since adults and youth showed significant differentiation. Metadata factors analyzed in each age group had to contain a sample size large enough to use in both adults and youth to allow for comparison. Because of this, some factors, such as having periodontal disease, were not analyzed since no youth had the disease. Genera percent coverage is represented as the percent of samples in which a genus was detected and was only calculated if a genus was present in at least 75% of samples in either age group. Genera coverages and abundances were calculated for each age group based on the rarified read counts to allow comparison with the diversity analyses. To determine the impact of familial relationships on oral microbiome beta diversity, unweighted and weighted UniFrac distances between participants of the same family were compared against participants from different families. A similar grouping was performed to determine mother-child distance comparisons between and within families. Benjamini/Hochberg FDR *p*-value adjustment was performed to account for multiple comparisons between participants of the same family.

### Statistical analyses

Our overall objective was to use a crowdsourcing model to obtain oral bacterial composition data to determine oral microbiome associations with an individual’s demographics, lifestyle, and/or genetics. An N of 351 (n_adults_ = 172 and n_youth_ = 179) used in this study is one of the largest used in a study of its kind to the best of our knowledge. Estimating power for microbiome analyses can be challenging because the relationship between microbiota community structure data and each feature, which by nature is highly dimensional and non-parametric, and within-group distances are often unknown (e.g. beta diversity comparisons can involve the use of distance matrices containing 10,000 + distances). Because of this, beta

diversity comparisons in our study can detect extremely small effect sizes (f < 0.1) with a

statistical power ≥ 0.99 and estimated effect sizes of f ≅ 0.02 at the minimum recommended power of 0.8^76^. Alpha diversity metrics between adults and youth detect effect sizes of f = 0.15 at a power of 0.8^76^. Therefore, we believe we have adequate power to examine the effects of factors structuring the oral microbiome. Power analyses were performed using the R statistical software pwr package version 1.2–2^77^.

Alpha (H, J, richness, phylogenetic richness) and beta (UniFrac) diversity metrics were tested against sample metadata factors using the QIIME2 diversity plugin^67^. Categorical metadata alpha diversity metrics were compared with pairwise Kruskal-Wallis tests using Benjamini/Hochberg FDR *p*-value adjustment for pairwise comparisons. Numeric alpha diversity metrics were compared with Spearman’s rank correlation. Categorical metadata beta diversity metrics were compared with pairwise permutational multivariate analysis of variance (PERMANOVA) tests using 999 permutations and Benjamini/Hochberg FDR *p*-value adjustment for pairwise comparisons. Numeric beta diversity metrics were compared with a two-sided Mantel test to identify Spearman’s rank correlation between distance matrices using 999 permutations. Family comparisons were tested with Wilcoxon rank-sum tests using Benjamini/Hochberg FDR *p*-value adjustment for multiple comparisons using the R statistical software stats package version 3.5.2^77^.

Analysis of composition of microbiomes (ANCOM) was used to identify features that were differentially abundant across significant demographic and health related groups (e.g. weight status, flosses, etc.) using the QIIME2 composition plugin^67,78^. ANCOM accounts for compositional constraints to reduce false positives when detecting differentially abundant genera. ANCOM W-statistics represent the strength of the ANCOM test and the F-scores represent the effect size of the feature. A high W-statistic with a high F-score would represent a feature that has strong statistical differentiation and is imparting a large effect difference on the data groups. Additionally, statistical comparisons of health attributes, liker status, and demographics were compared between adults and children with chi-squared tests using the SAS/STAT statistical software version 9.4 from SAS Institute Inc. Significance determined by *p* or *q* < 0.05.

## Supplementary information

Participant Metadata.

## Data Availability

Sequencing data and sample metadata are available on the QIITA open-source microbiome study management platform under study 11293 and EBI study ERP115887.
